# Framework for End-User Programming of Cross-Smart Space Applications

**DOI:** 10.3390/s121114442

**Published:** 2012-10-29

**Authors:** Marko Palviainen, Jarkko Kuusijärvi, Eila Ovaska

**Affiliations:** 1 VTT Technical Research Centre of Finland, P.O. Box 1000, FIN 02044 Espoo, Finland; 2 VTT Technical Research Centre of Finland, P.O. Box 1100, FIN 90571 Oulu, Finland; E-Mails: jarkko.kuusijarvi@vtt.fi (J.K.); eila.ovaska@vtt.fi (E.O.)

**Keywords:** end-user programming, cross-smart space applications, application framework

## Abstract

Cross-smart space applications are specific types of software services that enable users to share information, monitor the physical and logical surroundings and control it in a way that is meaningful for the user's situation. For developing cross-smart space applications, this paper makes two main contributions: it introduces (i) a component design and scripting method for end-user programming of cross-smart space applications and (ii) a backend framework of components that interwork to support the brunt of the RDFScript translation, and the use and execution of ontology models. Before end-user programming activities, the software professionals must develop easy-to-apply Driver components for the APIs of existing software systems. Thereafter, end-users are able to create applications from the commands of the Driver components with the help of the provided toolset. The paper also introduces the reference implementation of the framework, tools for the Driver component development and end-user programming of cross-smart space applications and the first evaluation results on their application.

## 1. Introduction

In the future there will be more and more smart spaces that enable users to share information, to monitor their environment and to control it. Therefore, smart space applications require at least three kinds of capabilities: controlling capabilities, monitoring capabilities, and information sharing capabilities are needed to assist the everyday life of the users of smart spaces. We believe that the future applications are *cross-smart space applications* that combine the resources and services that exist in various smart spaces to serve the needs of the user. For example, consider a case in which a consumer has a smart phone and a car that has an entertainment and navigation systems. In this case there could be two smart spaces: (i) the smart phone that provide context-sensing capabilities and (ii) the car that provide entertainment and navigation capabilities for the passengers. When the user is in the car, the cross-smart space applications assist (s)he to that take advantage of the possibilities of the available smart spaces. For example, there can be cross-smart space applications that use the context-sensing capabilities of the smart phone for controlling the entertainment and navigation systems of the car or use the navigation system of the car for controlling the smart phone.

Smart phones and small touch screen enabled mobile devices are very popular today. Mobile devices are always with the user and have smaller screen sizes than desktop computers when comparing both the physical size and the resolution. In addition, nowadays mobile devices are often touch screen devices, where the user controls the device with his/her fingers. It is not possible to provide ready-made cross-smart space applications for all possible needs but it should be possible for a person entering to a smart space to compose an application matching his/her needs *on-the-fly* in his/her mobile device and then to execute the application via his/her mobile device. The programming-by-example [1], visual programming [2], script-based creation [3], repository-based creation [4], and tailoring of applications techniques [5] are introduced for end-user programming. Unfortunately, these techniques are not designed for end-user programming of cross-smart space applications. Furthermore, these techniques are not applicable for end-user programming that is performed in a small touch-screen enabled mobile device. Therefore we decided to develop a novel end-user programming approach for cross-smart space applications.

In this paper a smart space is understood to be a Semantic Information Broker (SIB) that provides a named search extent for information. End-users can be professional programmers, software enthusiasts or non-programmers. This paper focuses on end-users that are non-programmers, use smart spaces via their mobile devices and are interested in creating new applications for their personal needs and daily tasks. The main research question is: how to support end-user programming of cross-smart space applications so that even non-programmers can create applications for their needs. Our solution to this problem is a framework that provides components for the creation and execution of cross-smart space applications. There are two novel points in the approach: (i) First, Semantic End-User Application Programming Interfaces (S-APIs) support creation of the execution flows and data flows of applications. (ii) Second, the execution components are ready-made building blocks that facilitate easy creation of cross-smart space applications that are independent from the smart space configuration. An S-API defines commands for the capabilities provided in the APIs of existing software system(s) and component(s) and inputs, outputs, and execution branches for each command. In our approach it is the commands' concern to control the execution flow. Thus, the “if” is implemented inside a command whereas the different execution branches define the “then” branches. Thus, the end-user does not need to use separate if-else structures in programming but the commands also specify the execution branches, to which the commands following the execution branch are attached. Furthermore, the ontology-based typing of the inputs/outputs of commands is used to guide the users to define only legal input/output connections between commands. The execution components automatically connect to the selected smart spaces, allocate capabilities for the application, execute the application, and free the allocated resources for the usage of other applications. Thus, the cross-smart space applications are not created for a fixed/specific set of smart spaces but are independent from the smart space configuration.

The Driver components are prerequisite for the use of the approach. Thus, the development of Driver components for various kinds of software systems/components should be easy for software professionals. This paper presents how much effort it required to implement Driver components for a Smart Phone and for a Home Automation System. Although the tools of the reference implementation greatly facilitated the implementation of the Driver components, it took three working days to implement these Driver components in our experiments. Thus, there is a still need for methods/tools that make the development of S-APIs/Driver components more effective. The mobile users require smooth and responsive applications, which presume a fast enough execution environment. Therefore we tested the performance of the used execution environment and observed that the execution speed of a cross-smart space application greatly depends on the used SIB implementations. In the experiments, the average execution time for a command was less than 2 ms for an internal Java-SIB, a 75-fold increase for an external C-SIB and an 800-fold increase for an external Java-SIB.

After this introduction, Section 2 introduces the related work and background of the framework presented in Section 3. The reference implementation for the framework is presented in Section 4. Our findings and experiences are discussed in Sections 5 and 6. Finally, conclusions are drawn in Section 7.

## 2. Related Works and Background

### 2.1. Terminology

The terminology used in this paper is presented in Table 1. The terminology consists of generic terms, Smart-M3 [6] and Interoperability Platform (IoP) architecture-specific terms [7], and terms that relate to our End-User Programming (EUP) framework.

### 2.2. End-User Programming Methods

The objective of the end-user programming methods is to bridge the gap between usage and programming of an application [8,9] and often these methods focus more on reusing of legacy software than creating new software components or source code. For example, the simplicity, support for immediate feedback and avoidance of misleading appearances are important in end-user programming tools [10]. The following briefly introduces end-user programming approaches taken from [4,9,11]:

1. *Programming-by-example*—Using a particular instance of execution, input-output relations, or existing programs as basis for creating new programs [11]. For example, modification of a working example speeds up development as it provides stronger scaffolding than writing code from scratch [1].
2. *Visual programming*—Replacing the textual programming notation with a graphical one with blocks and connectors [11]. Visual programming concepts and tools assist the user to create small applications on top of their things [2].
3. *Script-based creation*—Makes programming easier and more natural for users who want customized applications and are capable of doing basic programming without having to set up e.g., C++ or Java environments [11]. Script languages sacrifice execution efficiency and provide an interpreted development environment, a higher abstraction level for programming than typical system programming languages, and weaker typing than system programming languages [3].
4. *Repository-based creation of applications*—Supports the reuse of software components. For example, the [4] presents an end-user programming approach for Web applications that consist of a (i) Pattern library, (ii) Pattern language, and (iii) Command language. The Pattern library contains patterns (e.g., for dates, times, phone numbers, email addresses, and URLs), parsers (e.g., an HTML parser), and wrappers for Web sites such as Google or Amazon. The library patterns can be glued together with a pattern language called text constraints, which uses relational operators such as before, after, in, and contains to describe a set of regions in a page. The Tcl scripting language is used as a command language. The commands take patterns as arguments to indicate how to manipulate a Web page.
5. *Tailoring of applications*—Can be based on *customization* that modifies the parameters of components; *integration* that creates or modifies assemblies of components; and *extension* that creates new components by writing program code. The direct activation technique also belongs to this category and requires that the tailoring functionality is accessible from the use context when the need for tailoring occurs [5]. For example, the FreEvolve platform [12] provides an API for integrating tailoring functionality with software components that allows non-programmers to tailor an application by reassembling components at run-time visually [5].

Existing end-user programming methods are mainly based on abstraction; the goal is to hide details and to make the programming easier for non-programmers. Unfortunately, these methods are not designed to be used in small touch-screen enabled mobile devices. Therefore, this paper presents a novel end-user programming approach that supports visual programming, script-based and repository-based creation, and tailoring of applications. Firstly, the visual programming and scripts are used in the creation of applications. Secondly, the repositories support reuse of command sequences in end-user programming. Thirdly, the approach supports tailoring of applications in the following way; It is easy for an end-user: (i) to customize the parameters of the commands of the existing applications, (ii) to integrate the capabilities of Driver components to form new applications, and (iii) to extend available applications for new purposes. Applications can be downloaded from a smart space, modified/reconfigured with the provided tools, and thereafter, the modified applications can be executed in the smart environment.

### 2.3. Smart Space Architectures

The target of GLObal Smart Space (GLOSS) is to enable the interaction amongst people, artifacts, and places while taking into account of both context and movement on a global scale [13]. In the Web of Things vision, the physical world becomes integrable with computer networks so that the embedded computers and visual markers on everyday objects allow things and information about them to be accessible in the digital world [14]. Existing agent-based and/or service-based smart space application architectures (e.g., [15–18]) facilitate the development of smart space applications. An approach [19] that uses an Event-Condition-Action (ECA) service to perform event composition, event aggregation, and action execution actions on behalf of a client application is proposed, too. Furthermore, there is developed an architecture for a modular Event-Condition-Action (ECA) interaction model that facilitates the integration of different types of objects in a smart space, giving the user full control of their capabilities and facilitating creative mashuping to build customized functionalities that combine physical and virtual actions [20].

Two approaches (at least) are used for abstracting the diversity of the digital world: Ambient Networks and Semantic Information Brokers. The Ambient Networks are based on federations of multiple, cooperative networks that integrate and interwork the capabilities of different networks and abstract the inherent diversity, presenting an end-to-end and seamless network to a user [21]. The HYDRA-middleware can be seen to be an example of an Ambient Network approach that facilitates developers to create ambient intelligence applications based on the wireless devices and sensors [22]. This paper focuses on the Semantic Information Broker-based abstraction, in which separate agents communicate and share RDF triplets via a common database. For example, there is proposed an HTTP interface [23] that enables resource constrained Internet-enabled objects to communicate via common triple spaces. The framework described in this paper is based on the Interoperability Platform (IoP) [7] that aims at making the information in the physical world universally available to various services and applications, regardless of their location, which aligns well with the GLOSS and with the Web of Things vision, too.

In the IOP the key enabler for interoperability is the seamless exchange of information [7]: Information is exchanged without loss of meaning, among different applications running on different devices in any physical space. The IOP supports cross-domain interoperability (*i.e.*, the interconnection and communication between different technological platforms, possibly developed within different application domains) and cross-industry interoperability (*i.e.*, technical interoperability issues, such as commercial strategies, licenses, and regulations) by shifting the focus from the physical/service level to the information level. The IOP architecture follows the *blackboard* architecture and provides a cross-domain search extent and *publish-subscribe* paradigm for smart space applications that consists of two kinds of agents: Semantic Information Brokers and Knowledge Processors (KPs) [7]. A SIB is a lightweight Resource Description Framework (RDF) [7,24] database that takes care of information storing, sharing and governing and provides add, remove, query and subscribe functions for the KPs and for the semantic information stored to the SIB. Smart-M3 [6] and RIBS (RDF Information Broker Service) [25] are diverse implementations of the SIB intended for resource-rich and resource-scarce execution environments. KPs produces and/or consumes semantic information in a SIB and ontologies specify the meaning for the information [6,7].

The architectures listed in this subsection support the development of smart space applications but do not support the development/execution of cross-smart space applications. In many cases a smart space application cannot just be based on a SIB and communicating KPs but requires multiple SIBs. For example, there may be a need for an application that uses the personal SIB of a smart phone and the shared-SIB of a smart building. This paper extends this previous work and introduces an architecture/framework that enables end-user programming of cross-smart space applications. The novel point of our approach are the execution components that automatically connect to the selected smart spaces, allocate capabilities for the application, execute the application, and free the allocated resources for the usage of other applications. Thus, the applications are not created for a fixed/specific set of smart spaces but are independent from the smart space configuration.

### 2.4. Smart Space Application Development

**Figure 1.**
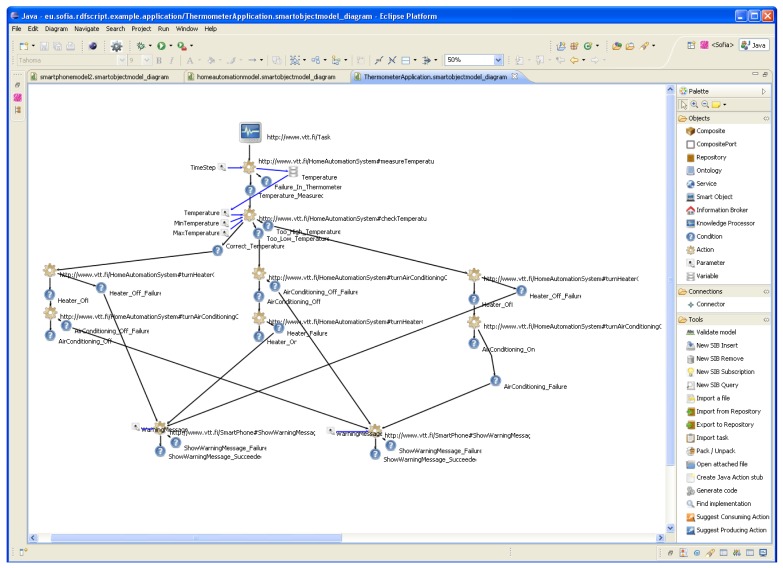
The main window of the Smart Modeller.

We have previously developed a tool, called *Smart Modeller* (shown in Figure 1) for the visual modelling of smart space applications. The tool is described in more detail in [26,27]. The Smart Modeller provides an extension point for extensions that are Eclipse plug-ins and perform processing related to the smart space application models. We decided to use the Smart Modeller as a starting point and to extend it to provide tool support for our end-user programming approach.

## 3. A Three-Layer Framework for End-User Programming of Cross-Smart Space Applications

**Figure 2.**
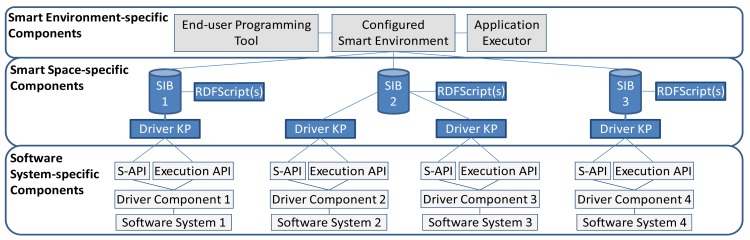
A three-layer framework for end-user programming of cross-smart space applications.

Our target was to develop an IoP-based architecture/framework (in Figure 2) to assist in end-user programming of cross-smart space applications. The IoP does not directly support creation of cross-smart space applications but it is KPs' concern to join to different SIBs and to transport the required semantic information between SIBs. Thus, in order to make the creation of applications more straightforward, we decided to develop an end-user programming framework for cross-smart space applications. The following paragraphs list the specific features of the framework:

### Two-level workflow for the creation of cross-smart space applications

**Figure 3.**
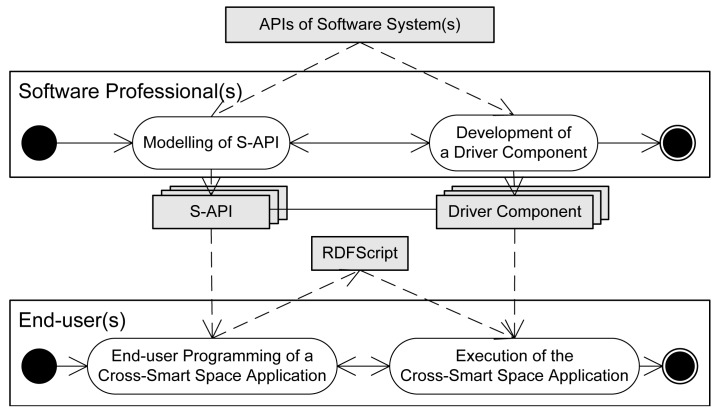
A two-level workflow for the creation of cross-smart space applications.

The framework enables the creation of applications in two integration steps (in Figure 3): (i) Software professionals focus on detailed programming tasks, model S-APIs and develop easy-to-apply Driver components for the APIs of existing software systems. (ii) Thereafter, end-users are able to create executable cross-smart space applications from the commands of Driver components with the help of the provided toolset.

### Execution components

End-user does not need to explicitly describe the SIBs used in the application but it is the execution components' concern to dynamically allocate resources for the application and to transport the required information between SIBs.

### S-APIs

**Figure 4.**
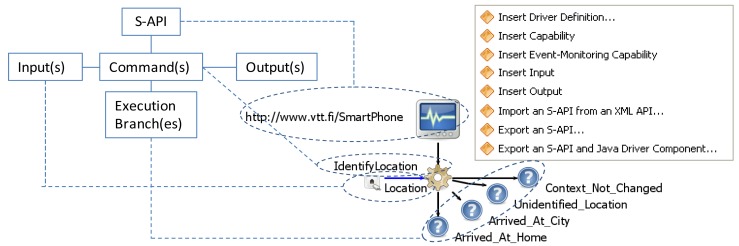
The Smart Modeller is used for the modeling of S-APIs.

Research on natural programming shows that end-users solve programmatic tasks using familiar logical constructs like “if” and “when”, combined with consequence words like “then” or “and” to specify and sequence procedures [10]. Mathematical operations and looping actions are avoided [28]. An S-API describes both the (i) *commands* for the capabilities provided in the APIs of existing software system(s) and component(s), and the (ii) *execution branches* (e.g., *command_ok* and *command_failed*) to which the commands following the execution branch can be attached. Thus, the end-user does not need to use separate if-else structures in programming but it is the commands' concern to control the execution flow. Thus, the “if” is implemented inside a command whereas the execution branches define the “then” branches. Figure 4 depicts the structure of an S-API: The S-API describes a unique identifier (URL), type (method or event-monitor) and inputs, outputs, and execution branches for each command. A name and type and possibly a default value are defined for each input of a command. The output defines a name and type for the data produced by a command.

### RDFScript language

It is a far too complex task for many end-users to compile executable applications from (e.g., Java or C++) source code. Thus, we decided to develop an interpretable script language, called RDFScript for end-user programming of cross-smart space applications.

The framework consists of three layers (seen in Figure 3) that are described in the following paragraphs:

### The layer of system-specific components

Execution of the commands of S-APIs is based on the Driver components. Smart space applications are often developed for physical environments that provide shared computing resources (e.g., mobile computers, embedded devices, and wireless networks) for end-users and their applications. These kinds of dynamic environments require that the Driver components must also take care of resource allocation and thus hide the complexity of execution from end-users.

### The layer of smart space-specific components

**Figure 5.**
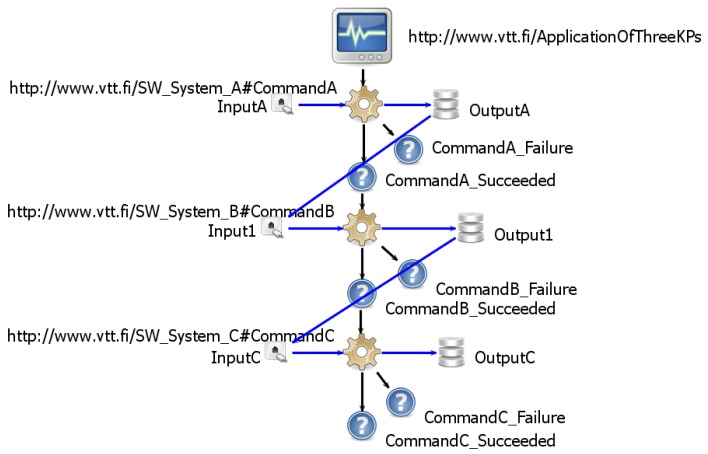
A cross-smart space application that uses the commands of S-APIs A, B, and C.

This layer consist of SIBs, Driver KPs, and RDFScripts. The *Driver KP* connects a Driver component to a smart space: It joins to the selected SIB, publishes the S-API of the Driver component to the SIB, and then waits allocation and execution requests from applications. The Notation3 (N3) language is a textual syntax alternative to RDF/XML [29]. For example, Appendix 1 shows the RDFScript application of Figure 5 in N3 format. The RDFScript can be published in N3 format to a SIB. Thus, SIBs are used as a deployment channel for smart space applications. In SIBs there can be multiple RDFScripts for a same purpose. Now, it is the user's concern to select the RDFScript that (s)he wants to use. However, more advanced selection mechanisms are needed to assist the user to pick up a correct RDFScript for a specific use case.

### The layer of smart environment-specific components

This layer consists of the Configure Smart Environment, End-user Programming Tool, and Application Executor components. The following subsections discuss these components in more detail.

### 3.1. Configured Smart Environment

The configured smart environment specifies smart spaces that are used in end-user programming and in execution of cross-smart space applications. The smart environment can consist of two kinds of SIBs:

1. *Internal SIBs*—The Application Executor uses a SIB via a KP Interface (KPI). The internal SIB can be a local component (e.g., a Java component) that implements the KPI. Thus, the usage of the KPI of an internal SIB does not require the usage of network connections and SSAP messages, which greatly speeds up execution of a cross-smart space application. Thus, for the performance and security reasons it may be better to use internal SIBs for the capabilities that are available in the user's personal device (e.g., in a mobile phone). Of course, these capabilities are available only for Application Executors that can connect to the internal SIB.
2. *External SIBs*—The external SIBs enable sharing of capabilities between different users in smart spaces. Unfortunately, the usage of external SIBs requires network connections and SSAP messages and thus limits the performance.

The RDFScript can optionally specify predefined smart spaces for application's execution or then an end-user can select smart spaces for application's execution. The execution data is visible for those KPs that are connected to the chosen smart spaces. Thus, in order to improve performance and security, it is better to use only a subset of smart spaces (e.g., more reliable and trusted smart spaces) in execution. This limits the risk that some harmful KPs spy the execution flow and data flow of a cross-smart space application. However, the selection of smart spaces is not the focal point of this paper but here we just assume that correct smart spaces are selected to be used in end-user programming and in execution of cross-smart space applications.

### 3.2. End-User Programming Tool

End-user programming of cross-smart space applications is performed in the end-user programming tool that takes the available S-APIs as an input and assists the end-user to specify a control flow and data flow for his/her application. As a result, it is easy for the end-user to create RDFScript applications for new purposes and to insert new commands to the command sequences of existing RDFScript applications. The end-user only has to choose an execution branch (see Figure 4), connect a new command to it, and finally configure the command's inputs by giving *absolute values* for inputs or by connecting inputs to have the values of the outputs of the previous commands of the sequence. The execution components will automatically distribute the input/output data between commands that are executed in various Driver KPs. Furthermore, the types of the inputs/outputs of commands are used to guide the users to define only legal input/output connections between commands.

### 3.3. Application Executor

The *Application Executor* is the central entity that controls the execution cycle of a cross-smart space application and uses the Execution APIs of Driver KPs via SIBs for coordinating the processing activities in the Driver KPs. It (i) interprets the RDFScript and (ii) joins to the selected smart spaces, and (iii) executes the application. The application execution consists of the query, allocation, execution, and recovery states. The following paragraphs describe these four states in more detail:

#### Query state

The Application Executor publishes a refresh capabilities request to the SIBs that deliver the request for the Driver KPs that will now publish descriptions about their capabilities to the SIB(s).

#### Allocation state

The Application Executor uses the Execution APIs, selects the best possible capabilities for the application and publishes allocate capability requests for the selected capabilities to the SIB. The Driver KPs must now share their resources for the application and publish responses for the allocate capability requests. The execution is continued if the required capabilities are successfully allocated for the application.

#### Execution state

The Application Executor publishes an execution request to the SIB that defines an identifier and inputs for a command. The Driver KP processes the request and publishes an execution response to the SIB. The Application Executor continues to the execution branch that is defined in the execution response and publishes an execution request for the next command. The input/output data is automatically distributed between commands that are executed in various processing nodes. At the same time, the resources that are not needed anymore in the application are freed for the usage of other applications. The execution is continued until the last command in the command sequence is executed. In the case of an event-monitoring command, the execution is paused until the activated event-monitor delivers an event to the SIB. The execution is then continued to the execution branch that is defined in the event.

#### Recovery state

The nature of a smart environment is very dynamic and both the Application Executors and Driver KPs can disappear (e.g., crash, are disconnected from the network or run low in batteries) at any time. Thus, it is important to handle these exceptional situations. For this reason there is defined a value for the maximum delay between commands. A Driver KP can request the Application Executors to publish their status to the SIB and then free those resources that are allocated for (e.g., crashed) applications that do not update their state to the SIB. Similarly, if a Driver KP does not deliver a response in time, the Application Executor cancels the execution of the application. The application state is removed from the SIBs after the application is executed or cancelled.

## 4. Reference Implementation for the End-User Programming Framework

The reference implementation of the framework supports the S-API/Driver component development in the desktop environment and end-user programming of cross-smart space applications that is performed either in the desktop environment or in the mobile environment or partially in both. The following subsections describe the execution environment and supporting tools in more detail.

### 4.1. Enhanced Execution Environment

Appendix 2 describes the communication (subject-predicate-object) triplets that are used in execution of RDFScript applications. The basic communication of Application Executors and Drivers is based on a very simple communication protocol that contains four kinds of messages: Driver Requests, Driver Responses, Application Executor Requests and Application Executor Responses. The Application Executors and Driver KPs have First-In, First-Out (FIFO) queues for these messages. The messages are stored to the queues and handled later in Application Executors and Driver KPs. The reference implementations of Application Executor and Driver KP are written in Java. The following techniques are used for improving the execution speed of cross-smart space applications:

#### A single triplet Driver requests and responses

The object of the triplet is a literal that specifies the content of the request/response message in an XML format. Thus, the number of triplets that must be subscribed from SIBs or published to SIBs and later removed from the SIBs is minimized. The application executor just needs to subscribe *ApplicationId RDFScript:DriverResponse* “” triplet whereas the Driver KP has to subscribe *DriverId RDFScript:DriverRequest* “” triplet. This shortens the initialization time of a cross-smart space application and decreases the processing related to the subscriptions in a SIB. Furthermore, the subscriptions are not needed to do during execution of commands but only when application execution is initialized.

#### Optimistic allocation for functional capabilities

In the first version of the framework, the Driver KPs were always requested to publish their functional capabilities to the SIBs and then the functional capabilities were allocated for the application. Now it is the Driver KPs' concern to publish descriptions of those functional capabilities that are not exclusively allocated for other applications to the SIB. The Application Executor tries then to allocate these functional capabilities for the application. However, if the Driver KP is crashed, there can be descriptions for functional capabilities that are no longer accessible via the SIB. If there are Driver KPs that do not respond to the allocation request, the Application Executor sends a refresh functional capabilities request, waits for responses, and then tries to allocate the resources for the application.

#### Blocking of event flooding

The Application Executor removes the event notification from the SIB after it has handled it. And the Driver KP does not publish a new event notification to the SIB before the Application Executor has removed its last event notification from the SIB. Thus, new event notifications are not published to the SIB until the Application Executor has handled the previous event notifications. This prevents the event-monitoring commands of Driver KPs to block the Application Executor that has not yet processed the previous events.

#### Garbage collection

The communication triplets used in the allocation and execution states of an application have the same subject (*i.e.*, *ApplicationId*). Thus, it is easy to recognize and remove these communication triplets from the SIBs after the application has stopped its execution.

#### S-APIs are published only when requested

In the execution phase only functional capability descriptions are published to SIBs. The entire S-API is published to SIBs only when needed, which decreases the initialization time of Driver KPs and the number of triplets stored to the SIBs.

#### Short encoding for the URLs used in the communication triplets

The properties/classes used in the communication triplets are represented by a single character (e.g., *RDFScript:refresh* is represented as *http://RS#A*) in order to decrease the amount of data delivered between Application Executors, Driver KPs, and SIBs.

### 4.2. Tools for the S-API/Driver KP Development

We extended the Smart Modeller tool to assists the development of S-APIs/Drivers and end-user programming of smart space applications. In order to improve usability, we decided to modify the Smart Modeller so that its extension can also be a toolset, *i.e.*, a dynamically created composite of tools that are specially selected and initialised for the model element(s) that the user has selected in the tool. Subsequently, we implemented separate toolsets for the S-API/Driver development and for end-user programming of cross-smart space applications.

Figure 4 presents an S-API for a smart phone that provides an *IdentifyLocation* command that takes a location as an input and output branches for identified location contexts (e.g., *Arrived_At_Home* and *Arrived_at_City*). The S-API is created by using the Smart Modeller and S-API/Driver development toolset that is used through a popup menu that is depicted in Figure 4. The toolset provides editing tools (e.g., the *Insert Driver Definition* and *Insert Capability* tools) to assist in creation of S-APIs. The tools are capable of storing an S-API in N3 format and transforming the S-API to a Java implementation of the Driver component. The manual coding that implements the actual functionalities to the Driver component can be performed by using the tools and code editors of the Eclipse environment.

### 4.3. Tools for End-User Programming in Desktop Environment

**Figure 6.**
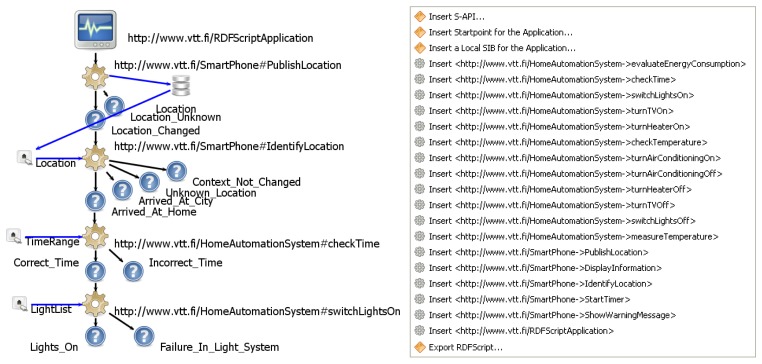
The Smart Modeller represents the RDFScript as a visual graph.

The creation of RDFScripts is based on the end-user programming toolset and on the Smart Modeller (Figure 6) that represents an RDFScript application as a visual graph that consists of elements and connectors. The black coloured connectors specify the execution flow and the blue coloured connectors specify data-flow for the RDFScript application.

The end-user programming is performed as follows. The end-user starts the Smart Modeller, creates a model for the application and then inserts a start point for the application (uses the *Insert Start Point for the Application* tool). The user will then use the *Insert S-API* tool and select S-APIs that (s)he want to use in the application. The repository elements that specify the S-APIs are now automatically inserted to the model. The *Element Importer* tool shows the commands of the S-APIs in a popup menu (in Figure 6) and thus enables the user to insert commands to the model. Subsequently, by drawing a connector between a condition and the inserted command element it is possible to attach the command to the command sequence. The user must configure the inputs of the command by defining a value for each input or then by connecting the inputs to the outputs of the previous commands in the command sequence. The user will finally use the *Export RDFScript* tool that stores the produced RDFScript in N3 format.

The RDFScript Simulator supports testing of applications/Driver KPs in the desktop environment. The user can first configure the smart environment and then insert new Driver KPs to the smart spaces of the smart environment. The user can select a Driver component that exists in the Java class path and start a Driver KP that connects the Driver component to a chosen smart space. The RDFScript Simulator also enables the user to upload applications to SIBs, download applications from SIBs, and finally execute applications in the configured smart environment.

**Figure 7.**
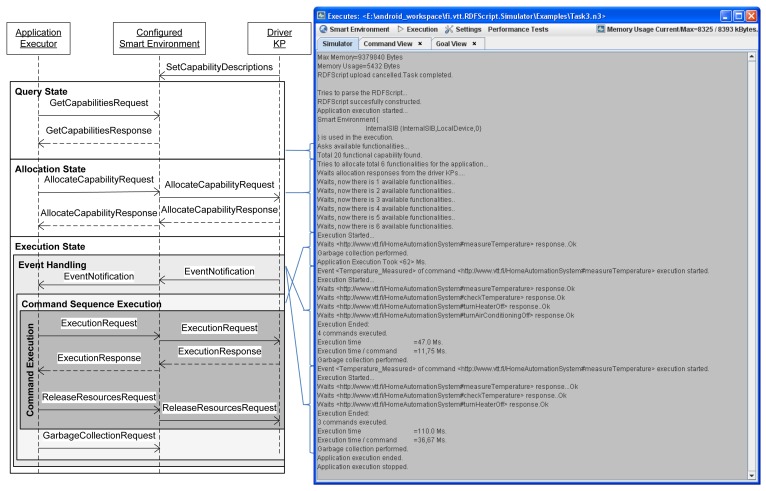
The execution of an application in the RDFScript Simulator.

Figure 7 presents a simplified sequence diagram for an execution sequence that is executed in the RDFScript Simulator. The sequence diagram shows the messages that are passed between an Application Executor, Configured Smart Environment and Driver KPs in the execution sequence, in which there is first queried capabilities, allocated capabilities for the application, executed the application and handled two event notifications.

### 4.4. Tools for End-User Programming in Mobile Environment

**Figure 8.**
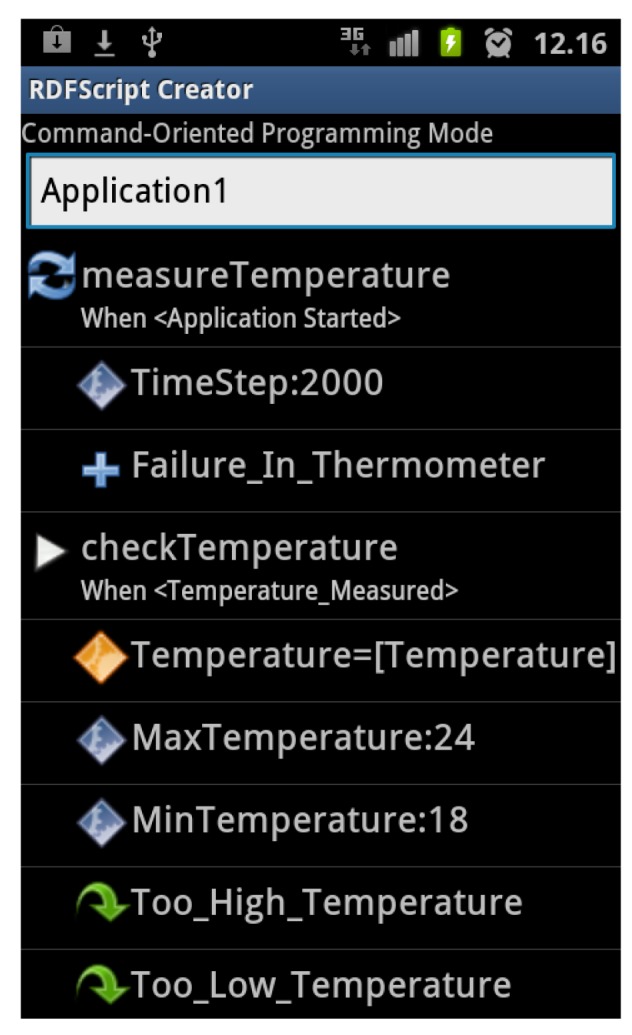
The end-user programming is performed in the list view of the RDFScript Creator.

It is difficult to create smart space applications via visual graphs (like in the Smart Modeller) in touch screen-enabled mobile devices. Thus, we decided to develop a new editor, called the RDFScript Creator (Figure 8) for the Android platform and for touch screen-enabled mobile devices.

**Figure 9.**
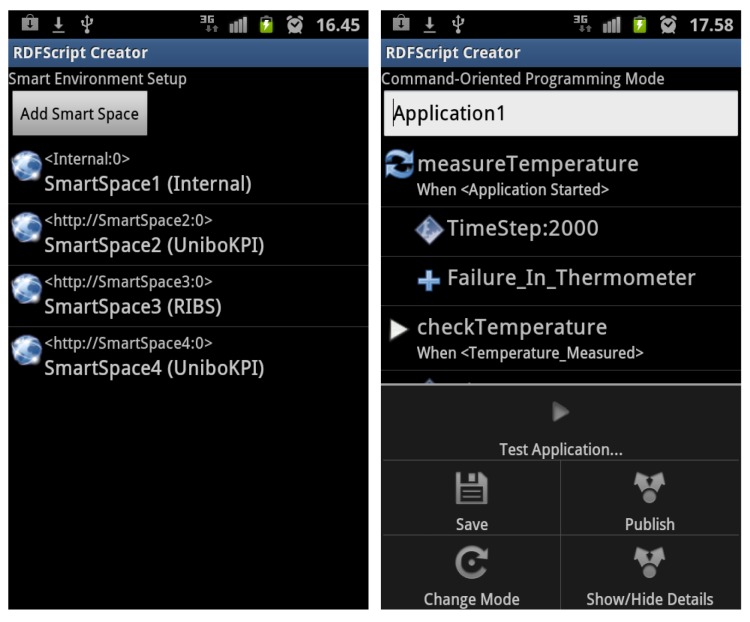
The cross-smart space application is executed in the configured smart environment.

The RDFScript Creator mostly provides the same features as the Smart Modeller in the desktop environment. The user can easily configure the smart environment in the smart environment setup view (Figure 9), download RDFScripts from the available smart spaces and save them for reuse, upload RDFScripts to smart spaces, and then execute cross-smart space applications in the configured smart environment. The main difference is the way how the application is created: Unlike in the Smart Modeller, the RDFScripts are not produced in a visual graph view but in a list view that represents RDFScripts as lists and sub lists (see Figure 8). The navigation between lists and sub lists is based on the links that enables to the user to navigate to the sub lists or to the upper-level lists. Thus, the user does not draw visual connectors between output branches and commands but uses the menus in the list view for creating these connections.

## 5. Evaluation

The objective of this section is to evaluate the applicability of the approach, implementation effort of S-APIs/Driver components, usability of the end-user programming part of the approach, and run-time performance of the execution environment.

### 5.1. Creation of S-APIs/Driver Components

The implementation of a Driver component typically requires a few hundred lines of coding. Of course, the implementation effort of the Driver component strongly depends on the software system for which the Driver component is implemented. In our experiment the creation of S-APIs took a few hours for the smart phone and for the home automation system. Then, skeletons of Driver components were generated from the defined S-APIs. The implementation of the Driver components took 3 working days. The sizes of S-APIs (in N3 format) and Driver components are depicted in Table 2. As can be seen, the Driver Component skeleton greatly assisted the developer in the implementation of the Driver components. The Driver component for the smart phone required 254 lines of coding and the Driver component for the home automation system required 244 lines of coding. Thus, the percentage of generated code was 47.7% for the Driver component of the smart phone and 63.0% for the Driver component of the home automation system.

The size of the Application Executor is 15,500 lines of code and the size of the Driver KP is 13,700 lines of code. The size of the compiled Java classes and interfaces of the Application Executor is 377 Kbytes and the size of the classes and interfaces of the Driver KP is 292 Kbytes.

### 5.2. Usability

We have got positive feedback from the end-users in experiments and it seems that the approach assists the non-experts to create applications for their own purposes. However, this paper does not focus on the usability issues but only briefly summarizes the main results of the usability test, introduced in detail in [30].

A small-scale usability test was performed by 8 programmers and 8 non-programmers. In the test, the participants had to compose an application that switches lights on, turns television on, checks temperature and finally turns air conditioning on, if the temperature is too high. Furthermore, the users performed a task, in which they had to modify a ready-made application that controls the heating/air conditioning system at home. The users added commands to the application that will show a message in the user's smart phone, if there is a failure in the heating or air conditioning system.

The tests showed that the framework facilitates the end-user programming that specifies a control flow and data flow for a cross-smart space application: Firstly, it was easy for the users to add commands to the execution branches of commands. Secondly, in order to facilitate the creation of data flows, the RDFScript Creator tool was enhanced so that it recommends commands that produce suitable input data for a selected command. As a result, a user can add a command and define its inputs manually or use automatic input values produced by other commands. For example, if the user decided to use the automatic temperature value in the *Check Temperature* command, the editor automatically added the *Measure Temperature* command (if needed) to the sequence and then connected its output to the temperature input of the *Check Temperature* command. This greatly improved usability and in later tests it was much easier for the users to perform the given end-user programming tasks.

In summary, both the programmers and non-programmers were quite satisfied with the end-user programming approach and it was very fast to learn: It took only a few minutes for the participants to learn to use the approach. Furthermore, the approach works well for developing relatively small applications that contain less than 20 commands. S-APIs/Driver KPs have a significant effect on the usability of the approach. Thus, end-user testing/feedback are necessary to ensure that the S-APIs are suitable for end-user programming. In addition, the following issues should be considered in the development of S-APIs/Driver KPs: Firstly, the S-API creator must choose the capabilities that are provided for end-user programming. Secondly, the end-user should find the correct commands for his/her application and thus it is important to give very descriptive names for commands/Driver KPs. Thirdly, the number of the inputs of commands should be minimized because it can be laborious to use commands that have a great number of inputs in the different kinds of applications.

### 5.3. Performance

In order to evaluate the performance of the execution environment we first created S-APIs for software systems A, B, and C. Each S-API defined only one command that takes an integer value as an input, then increases the value by 1, and returns the result as an output. Then we wrote three test cases that automatically composed command sequences of the commands A, B, and C of the S-APIs of the software systems A, B, and C. The size of the composed test sequences was between 1 and 100. We then executed these test sequences and measured how the length of a command sequence and the number of SIBs affect the average execution time of a command.

In the first test case only a single SIB and the command A of the software system A was used. Two SIBs were used in the second test case. The command sequence consisted of Command A, B, A, B, *etc*. calls so that the output of the previous command was delivered for the following command. The third test case used three SIBs. The command sequence consisted of Command A, B, C, A, B, C *etc*. call sequences (in Figure 6) so that the output of the previous command was delivered for the following command.

**Figure 10.**
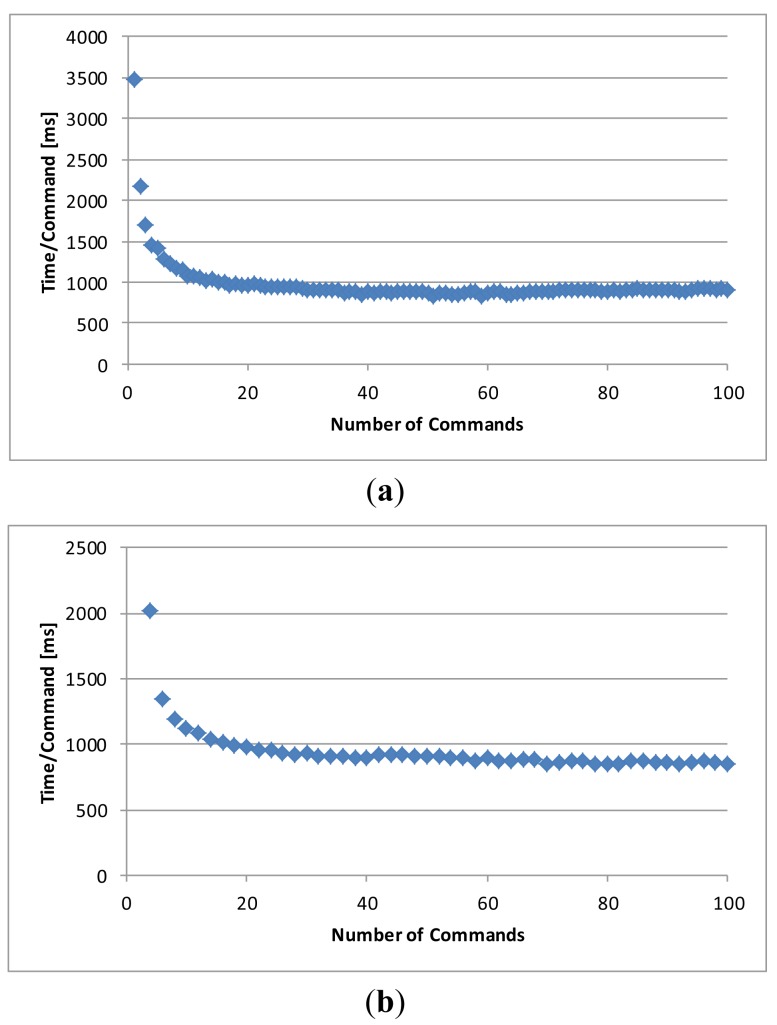

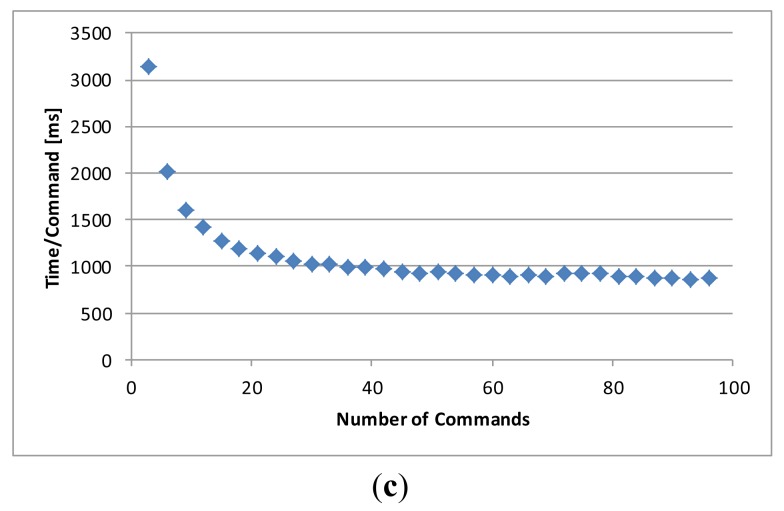
(**a**) The average execution time for a command in the test case 1 (one SIB and Driver KP). (**b**) The average execution time for a command in the test case 2 (two Driver KPs and SIBs). (**c**) The average execution time for a command in the test case 3 (three Driver KPs and SIBs).

The performance testing was performed by using external SIBs and socket connections and the RDFScript Simulator in a laptop computer (Dell E6500, Intel Core Duo CPU 2.66 GHz, 4 GB of RAM, and JRE 1.6.0_16). The average execution time for a command was measured for the three test cases (depicted in Figure 10(a–c)). As can be seen, the better performance is obtained in longer command sequences.

The main reason for this is the fact that a smaller part of the execution time is used for joining to the smart spaces and for the resource allocation/release activities. The network connections and SIB implementations will greatly affect the execution speed of a cross-smart space application. The used external SIB was implemented in Java but we have also used an external SIB that is implemented in C language. In this case the performance is greatly improved and the average execution time for a command was less than 150 ms in a test case 1 when the size of the command sequence was 10 commands. Unfortunately, this SIB implementation is on a prototype stage and we cannot yet utilize it in execution of wider cross-smart space applications.

In some cases it is possible to use internal SIBs. The execution time for a command (the size of the command sequence was 10) was less than 2 ms in a test that was executed by using an internal SIB and the RDFScript Simulator in the laptop computer. The average execution time for a command was about 850 ms in a test that used two external SIBs and socket connections in the simulator environment. The average execution time for a command was 1,600 ms when the same test was executed in an Android phone (Samsung Google Nexus S, 1 GHz Cortex-A8, and 512 MB of RAM) that used two external SIBs through WLAN connections.

The execution environment enables the sharing of RDFScript applications via SIBs. In the RDFScript Simulator it took 17.5 s to upload an RDFScript application that size was 17 Kbytes in N3 format to the external Java SIB. The downloading this same application from the external SIB took 14.8 s.

However, it is important to note that it is not possible to generalize the measured performance values for different kinds of execution environments but there are many issues that have effect on the execution speed of a cross-smart space application. Firstly, the performance of the used SIB implementations and network connections greatly affect the execution speed of a cross-smart space application. Secondly, the number of triplets stored to SIBs and the number of subscribed triplets from SIBs affect the execution speed of a cross-smart space application.

## 6. Discussion

The paper introduced a future-proof approach using standardized W3C technologies such as RDF and schemas, allowing the opportunity to extend the lifetime of deployed applications, as well as the enhancement of applications developed this way to encompass new smart spaces or information. The presented approach enables end-users to use the informational/computing capabilities of the APIs of existing software systems/components in their applications. A developer (e.g., an end-user) does not necessarily need to think the run-time configuration of KPs and SIBs that execute his/her smart space application. The ready-made execution components hide the execution architecture of KPs/SIBs, will automatically allocate resources for the application, transport the required data between commands, and finally orchestrate the processing activities in Driver KPs. Furthermore, the execution environment provides ready-made optimisations (a single triplet Driver requests and responses, short encoding for URLs used in the RDFScript, optimistic allocation for functional capabilities, blocking of event flooding, and garbage collection) for execution of cross-smart space applications.

Unfortunately, the following issues may limit the utilisation of the presented approach: Firstly, the use of the approach requires that the target software systems/components provide open APIs for developers. Secondly, the rapid deployment of new Driver components is a challenge and requires developers that want to implement S-APIs/Driver components for a wide variety of smart spaces and environments. An important motivation for this work is the fact that the S-APIs/Driver components produce additional value and can gain new users for existing software systems and components. Furthermore, the provided tools facilitate the S-API/Driver component development and thus lower the threshold of the implementation of these components. However, it took three working days to implement two Driver components in our experiments. Thus, there is a still need for methods/tools to speed up the development of the S-APIs/Driver components. Moreover, the business potential of the providers of APIs/Driver components needs also to be studied in the future.

The RDFScript language is designed for end-user programming and thus provides only limited expressiveness related to programming languages such as Java/C. For example, it does not offer if-else, while, for, and try-catch structures that exist in many programming languages.

The S-APIs have a significant effect on the usability of the approach and the end-user testing/feedback is required for ensuring that the S-APIs are suitable for end-user programming. The S-APIs typically do not provide access to all the capabilities that are provided in the APIs of software systems/components. However, it is possible to later develop extended versions of the S-APIs/Driver components that will better cover the capabilities of the APIs. The standardized and tested S-APIs could facilitate the development of Driver components. Device manufacturers could implement Driver components for these S-APIs and enable end-users to utilize their products in different kinds of applications. Furthermore, standardised data models could be developed for the input/output data used in the S-APIs. As a result, it is possible to describe commands that produce data for the commands of other S-APIs. However, instead of standard S-APIs and data models we believe more on incremental and application-driven development of the S-APIs/Driver components where a cross-smart space application is first sketched and thereafter S-APIs/Driver components are developed for it. The next cross-smart space applications will be then based on the existing S-APIs/Driver components. If needed, a few new S-APIs/Driver components are developed for the new application, by taking care that they are compatible with the existing S-APIs/Driver components.

Although the framework has been applied to the development of cross-smart space applications it has not yet been validated in an industrial context. Thus, more applicability validation of the approach is still needed. For example, there is a need for long-duration empirical tests to study how much the approach assists and speeds up end-user programming of applications that are based on the capabilities of different kinds of software systems. However, we believe that the fact that the approach promotes the use of capabilities of legacy software systems in end-user programming will motivate its industrial use. The prerequisite is that the software professional are familiar with the approach and are motivated to implement S-APIs/Driver components for end-user programming.

## 7. Conclusions and Future Work

This paper makes two main contributions: (i) a component design and scripting method for end-user programming of cross-smart space applications, and (ii) a backend framework of components that interwork to support the brunt of the RDFScript translation, and the use and execution of ontology models. Before end-user programming activities, the software professionals must focus on the more difficult tasks and develop easy-to-apply S-APIs/Driver components for the APIs of existing software systems/components. Thereafter, end-users are able to create applications from the commands of the S-APIs with the help of the provided toolset. This paper also describes a reference implementation for the framework and tools to support creation of the Driver components and end-user programming of smart space applications.

The target of the implemented execution components and tools just were to test the feasibility of the approach. Thus, although these implementations facilitate end-user programming of cross-smart space applications, more (usability) improvements are still needed for the execution components/tools. Our future goal is to utilize the execution components/tools in smart spaces that contain Driver KPs that are deployed to embedded devices, mobile devices, and desktop computers. Furthermore, our objective is to integrate the RDFScript environment with native GUI systems e.g., QML, Web-based, and Java/Python GUIs and explore end-user programming of cross-smart space applications for different purposes and by the different groups of end-users.

## Figures and Tables

**Table 1.** Terminology.

	**Term**	**Description**	
Generic Term	Capability	A potential or stated ability before execution	
	Resource	An actual used/available capacity during execution	
	Application Programming Interface (API)	The APIs of software systems/components typically contain methods that provide specific kinds of functionalities for their users and/or possibly event-monitors capable of delivering observed events for the observers.	
Smart-M3/IOP-specific Term	Semantic Information Broker (SIB)	A SIB is a lightweight (RDF) database that takes care of information storing, sharing and governing and provides add, remove, query and subscribe functions for the KPs and for the semantic information stored to the SIB.	
	Knowledge Processor (KP)	A KP produces and/or consumes semantic information in a SIB and ontologies specify the meaning for the information [6,7].	
	Smart Space Access Protocol (SSAP)	The communication between KPs and SIBs is based on the SSAP and semantic information that is described in the RDF format [6,7].	
End-User Programming (EUP) Framework-specific Term	Configured Smart Environment	A configured smart environment specifies the smart spaces to be used in end-user programming and in execution of cross-smart space applications.
	Command	A command provides a specific kind of functionality to be used in a cross-smart space application.
	Semantic End-User Application Programming Interface (S-API)	An S-API defines commands for the methods of APIs and commands to activate the event-monitors. The S-API describes a unique identifier (URL), type (method or event-monitor) and inputs, outputs, and execution branches for each command. A name and type and possibly a default values are defined for each input of a command. The output defines a name and type for the date produced by a command.
	Driver Component	A Driver Component implements the S-API and Execution API and contains six methods for obtaining (i) a unique identifier for the Driver and (ii) descriptions for its available capabilities and also methods for (iii) allocating capabilities for the usage of an application (iv) activating event-monitoring in the Driver, (v) execution of commands, and (vi) releasing the allocated resources.
	Execution component	Two kinds of execution components are used in the framework: (i) Application Executor and (ii) Driver KP components. The execution components automatically connect to the selected smart spaces, allocate capabilities for the application, execute the application and distribute the input/output data between commands that are possible executed in various processing nodes and different smart spaces, and finally release the allocated resources for the usage of other applications.

**Table 2.** The sizes of the implemented S-APIs and Driver components.

**Target System**	**The Size of S-API [Kbytes]**	**The Size of a Driver Component Skeleton [Lines of Code]**	**Coding Effort [Lines of Code]**	**The Size of a Driver Component [Lines of Code]**	**Percentage of generated code [%]**
Smart Phone	8.7	232	254	486	47.7
Home Automation System	18.6	415	244	659	63.0

## Appendix 1.

**Table:** The RDFScript application of Figure 5 in N3 format.

@prefix rdf: <http://www.w3.org/1999/02/22-rdf-syntax-ns#>	
@prefix rdfs: <http://www.w3.org/2000/01/rdf-schema#>	
@prefix model: <http://www.vtt.fi/opens/adk/metamodel#>	
@prefix diagram: <http://www.vtt.fi/opens/adk/diagram#>	
# http://www.vtt.fi/ApplicationOfThreeKPs null	
_:node rdf:type model:Graph; model:name “http://www.vtt.fi/ApplicationOfThreeKPs; rdfs:comment “not provided”;	
model:contains _:node_1, _:node_2, _:node_3, _:node_4, _:node_5, _:node_6, _:node_7, _:node_8, _:node_9, _:node_10, _:node_11, _:node_12, _:node_13, _:node_14, _:node_15, _:node_16, _:node_17, _:node_18, _:node_19, _:node_20, _:node_21, _:node_22, _:node_23, _:node_24, _:node_25, _:node_26, _:node_27, _:node_28, _:node_29, _:node_30, _:node_31, _:node_32, _:node_33	
_:node_1	rdf:type model:KnowledgeProcessor; model:name “http://www.vtt.fi/ApplicationOfThreeKPs diagram:x “545”; diagram:y “200”.
_:node_2	rdf:type model:Action; model:name “http://www.vtt.fi/SW_System_A#CommandA; model:implementation “method”; model:type “method”; diagram:x “563”; diagram:y “311”.
_:node_3	rdf:type model:Condition; model:name “CommandA_Succeeded”; model:logicExpression “DefaultValue”; diagram:x “565”; diagram:y “410”.
_:node_4	rdf:type model:Condition; model:name “CommandA_Failure”; diagram:x “625”; diagram:y “375”.
_:node_5	rdf:type model:Parameter; model:name “InputA”; model:position “1”; model:value “1”; model:type “http://www.exampleontology.com#DataClass;; diagram:x “463”; diagram:y “316”.
_:node_6	rdf:type model:Variable; model:name “OutputA”; model:value “”; model:type “http://www.exampleontology.com#DataClass; diagram:x “673”; diagram:y “316”.
_:node_7	rdf:type model:Action; model:name “http://www.vtt.fi/SW_System_B#CommandB;; model:implementation “method”; model:type “method”; diagram:x “560”; diagram:y “485”.
_:node_8	rdf:type model:Condition; model:name “CommandB_Succeeded”; model:logicExpression “DefaultValue”; diagram:x “565”; diagram:y “575”.
_:node_9	rdf:type model:Condition; model:name “CommandB_Failure”; diagram:x “630”; diagram:y “550”.
_:node_10	rdf:type model:Parameter; model:name “Input1”; model:position “1”; model:value “”; model:type “http://www.exampleontology.com#DataClass;; diagram:x “450”; diagram:y “490”.
_:node_11	rdf:type model:Variable; model:name “Output1”; model:value “”; model:type “http://www.exampleontology.com#DataClass;; diagram:x “705”; diagram:y “490”.
_:node_12	rdf:type model:Action; model:name “http://www.vtt.fi/SW_System_C#CommandC;; model:implementation “method”; model:type “method”; diagram:x “560”; diagram:y “670”.
_:node_13	rdf:type model:Condition; model:name “CommandC_Succeeded”; model:logicExpression “DefaultValue”; diagram:x “565”; diagram:y “770”.
_:node_14	rdf:type model:Condition; model:name “CommandC_Failure”; diagram:x “615”; diagram:y “740”.
_:node_15	rdf:type model:Parameter; model:name “InputC”; model:position “1”; model:value “”; model:type “http://www.exampleontology.com#DataClass;; diagram:x “455”; diagram:y “675”.
_:node_16	rdf:type model:Variable; model:name“OutputC”; model:value “”; model:type “http://www.exampleontology.com#DataClass;; diagram:x “680”; diagram:y “675”.
_:node_17	rdf:type model:Connector; model:relationship “model:triggers”; model:source _:node_1; model:target _:node_2.
_:node_18	rdf:type model:Connector; model:relationship “model:triggers”; model:source _:node_2; model:target _:node_3.
_:node_19	rdf:type model:Connector; model:relationship “model:success”; model:source _:node_2; model:target _:node_4.
_:node_20	rdf:type model:Connector; model:relationship “model:inputs”; model:source _:node_5; model:target _:node_2.
_:node_21	rdf:type model:Connector; model:relationship “model:produces”; model:source _:node_2; model:target _:node_6.
_:node_22	rdf:type model:Connector; model:relationship“model:triggers”; model:source _:node_7; model:target _:node_8.
_:node_23	rdf:type model:Connector; model:relationship “model:success”; model:source _:node_7; model:target _:node_9.
_:node_24	rdf:type model:Connector; model:relationship “model:inputs”; model:source _:node_10; model:target _:node_7.
_:node_25	rdf:type model:Connector; model:relationship “model:produces”; model:source _:node_7; model:target _:node_11.
_:node_26	rdf:type model:Connector; model:relationship “model:triggers”; model:source _:node_12; model:target _:node_13.
_:node_27	rdf:type model:Connector; model:relationship “model:success”; model:source _:node_12; model:target _:node_14.
_:node_28	rdf:type model:Connector; model:relationship “model:inputs”; model:source _:node_15; model:target _:node_12.
_:node_29	rdf:type model:Connector; model:relationship “model:produces”; model:source _:node_12; model:target _:node_16.
_:node_30	rdf:type model:Connector; model:relationship “model:triggers”; model:source _:node_3; model:target _:node_7.
_:node_31	rdf:type model:Connector; model:relationship “model:maps”; model:source _:node_11; model:target _:node_15.
_:node_32	rdf:type model:Connector; model:relationship “model:maps”; model:source _:node_6; model:target _:node_10.
_:node_33	rdf:type model:Connector; model:relationship “model:triggers”; model:source _:node_8; model:target _:node_12.

## Appendix 2.

**Table:** Communication triplets for execution of RDFScript applications.

**State**	**Message**	**Triplet(s)**	**Description**
**Query**	Refresh Capabilities Request	*ApplicationId* *RDFScript:refresh “”*	The Application Executor requests the Driver KPs to update their capability descriptions to the SIB.
	Capability Description	*DriverId#CommandId* *rdf:type RDFScript:Capability*	A Driver publishes descriptions about its capabilities to the SIB.
**Allocation**	Allocate Capability Request	*ApplicationId* *RDFScript:DriverRequest* * “XML Message”*	It is the Application Executor's concern to allocate required capabilities for the application.
	Allocate Capability Response	*ApplicationId* *RDFScript:DriverResponse* * “XML Message”*	It is the Driver KP's concern to share its resources for the application.
**Execution**	Execution Request	*ApplicationId* *RDFScript:DriverRequest* * “XML Message”*	The Application Executor interprets the RDFScript and publishes the execution requests to the SIBs.
	Execution Response	*ApplicationId* *RDFScript:DriverResponse* * “XML Message”*	The Driver KP publishes an execution response for the received execution request. Execution continues to the execution branch that is defined in the response.
	Event Notification	*ApplicationId* *RDFScript:DriverResponse* * “XML Message”*	The Driver KP monitors events and publishes the events to the SIB. Execution continues to the execution branch that is defined in the event notification.
	Release Resources Request	*ApplicationId* *RDFScript:DriverRequest* * “XML Message”*	It is the Application Executor's concern to free the allocated resources after they are not needed in the application.
**Recovery**	Refresh Application State Request	*DriverId* *RDFScript:refreshApplicationState* * “”*	This operation is used for the Driver KP recovery. The Driver KP requests the Application Executors to publish their states to the SIB.
	Application State Response	*ApplicationId* *RDFScript:applicationState* * “Active”*	When requested, the Application Executor must publish its state to the SIB. The Driver KPs free resources that are allocated for applications that do not update their states to the SIB.
